# What We Expect from the College of Nursing

**Published:** 1918-12-28

**Authors:** 


*To the Editor of* THE HOSPITAL.

### WHAT WE EXPECT FROM THE COLLEGE OF NURSING.

*To the Editor of* THE HOSPITAL.

SIR,—What do we expect the College of Nursing to accomplish for us? Why did we join? Because we have long felt the need of an organisation, and suffered much in consequence of its absence. We want, of course, State Registration. But first of all we want a decent wage—a wage to enable us to "live" and provide for sickness or a possible old age. We want independence. We want remuneration equal to services rendered. We want our profession acknowledged and treated as any other profession; we are out to make a living by it. We love our work; so does the doctor, the lawyer, etc., yet they expect, and justly so, to be paid in full for their services.

It is enough to make one turn in disgust from nursing and everything associated with it, when on the completion of a four years' training, after having first paid an entrance fee of from thirty to seventy guineas, with no salary for first two years and a nominal one of £10 and £14 respectively for third and fourth years, as is the case in a great many Irish hospitals, and has been so in my own case, we emerge triumphantly with our certificate, brimming over with knowledge, skill, and experience (dearly purchased, I assure you), to seek an appointment, and are offered from £30 to £35 yearly—wages that would insult a self-respecting charwoman. What remains for us to do but take it? To go on strike is considered degrading, and the delicate nature of our work prevents us from absenting ourselves even for one day. We cannot shout our grievances from the housetops; the public never read the nursing papers, and the hospital authorities never want to. Well, then, we expect the College to act as our spokesman and do the shouting, while we work in silence; that was our object in joining.

By all means let the College raise the standard—it is an excellent thing to do—but let it begin by having the trained nurses' salaries raised; and with improved conditions, better wages, and shorter hours the standard will do much towards righting itself, by attracting more women of education and standing to the ranks, and keeping those outside who are contented with the crumbs. Lastly, we want a badge, medal, or some mark of distinction, whereby a nurse may be easily recognised from an impostor who for some reason wishes to don her uniform; and the fact of members wearing such distinction would, in my opinion, induce others to join, and therefore swell the ranks of the College Register. Surely the College has had ample time to design something, even a brass button! As yet we have derived no benefit, but, with implicit confidence in our leaders, "we are waiting." E. DE M.

